# An algorithm for fragment-aware virtual network reconfiguration

**DOI:** 10.1371/journal.pone.0207705

**Published:** 2018-11-21

**Authors:** Xinbo Liu, Buhong Wang

**Affiliations:** Information and Navigation college, Air Force Engineering University, Xi’an, Shaanxi, China; University of Konya Technical, TURKEY

## Abstract

In view of the fact that the current online virtual network embedding algorithms do not consider the fragment resources generated in the embedding process deeply enough, resulting in the problem that the acceptance ratio and the revenue to cost ratio are both low, a mathematical model for virtual network reconfiguration is constructed and a heuristic algorithm for fragment-aware virtual network reconfiguration (FA-VNR) is proposed. The FA-VNR algorithm selects the set of virtual nodes to be migrated according to the fragment degrees of the physical nodes, and selects the best virtual node migration scheme according to the reduction of the fragment degrees of the physical nodes as well as the reduction of the embedding cost of the embedded virtual networks. Extensive simulation results show that the proposed FA-VNR algorithm not only can obviously improve the acceptance ratio and the revenue to cost ratio of the current online virtual network embedding algorithm, but also has better optimization effect than the existing virtual network reconfiguration algorithm.

## Introduction

Network virtualization allows multiple heterogeneous virtual networks (VNs) to share and run on the same physical network, which can solve the ossification problem of Internet [1–4]. However, network virtualization brings flexibility to the network architecture while also presenting new challenges. One of the major challenges in network virtualization is how to efficiently and effectively allocate physical network resources to the arriving VN requests, which is known as the virtual network embedding (VNE) problem.

In order to achieve network virtualization as soon as possible, in recent years, the VNE problem has mostly been of academic interest, and a lot of online VNE algorithms have been proposed [5–9]. Authors in [5] propose a VNE algorithm named VNE-DCC, whose acceptance ratio and revenue to cost ratio are attractive. The VNE-DCC algorithm considers not only the local resources of a node, but also the topological attributes of its neighborhood nodes. However, the VNE-DCC algorithm does not consider how to reduce the fragment resources generated in the physical network during the VNE process.

For the online VNE algorithms, the arrival time, resource demand, and lifetime of each VN request are random variables. With the arrival and departure of the VN requests, the resources of the physical network may be fragmented [10], and the embedding schemes for the VN requests that have been allocated resources on the physical network may become non-optimal [11]. These will cause the rejection of future VN requests. Therefore, in order to improve the performance of the online VNE algorithms, it is necessary to introduce the reconfiguration mechanism into the online VNE process, and dynamically adjust the node load and link load of the physical network.

Compared with online VNE problem, the VN reconfiguration problem is relatively less explored and only a few VN reconfiguration algorithms are proposed. Authors in [12] give a reactive VN reconfiguration scheme for the online VNE problem, where the VN reconfiguration algorithm is run only when a VN embedding strategy fails to admit a VN request. The main contribution of their work is the proposal of an integer linear programming (ILP) formulation for the reconfiguration. The simulation results show that the acceptance ratio can be significantly improved with reconfigurations. However, the ILP problem belongs to the NP-hard problem. When the instance of the problem is large, it is difficult to find the optimal solution within a limited time. In order to minimize the execution time of VN reconfiguration algorithms, some scholars use heuristic-based or metaheuristic-based solutions to solve the VN reconfiguration problem. Authors in [13] propose a heuristic VN reconfiguration algorithm, in which the physical link bandwidth utilization is driven to trigger virtual link migration. Authors in [14] propose a VN reconfiguration approach, which is validated in different network size and link density scenarios. The pivotal element of their approach is the virtual link migration problem, which is meant to find optimal reconfigurations of virtual links on the physical network in runtime. Authors in [15] propose a simulated annealing based metaheuristic algorithm to solve the VN reconfiguration problem. The objective of their work is to reduce the number of bottleneck physical links as well as the total bandwidth consumption of the embedded VNs. Although the above algorithms improve the acceptance ratio of VN requests, they all pay too much attention to the reconfiguration of virtual links, and not pay enough attention to the reconfiguration of virtual nodes. Author in [16] propose a reactive VN reconfiguration algorithm to improve acceptance ratio. The main idea of the VN reconfiguration is to reconfigure the current embeddings to obtain the physical network with as many low-stress physical nodes and links as possible. Authors in [17] propose a VN reconfiguration algorithm based on minimum cost. The algorithm improves the acceptance ratio of the VN requests and the load balancing degree of the physical network by migrating fewer virtual nodes and virtual links. Based on the literature [17], authors in [18] consider the close relationship between the virtual nodes in the virtual node migration process, and further improve the acceptance ratio of the VN requests.

In summary, the existing VN reconfiguration algorithms either emphasize too much on reconfiguring the bottleneck physical links, or overemphasize the reconfiguration of the bottleneck physical nodes. In this paper, in order to further improve the performance of the existing VNE algorithms, we consider the VN reconfiguration problem from two aspects: bottleneck nodes and bottleneck links. We first define a method to measure the fragment degrees of the physical nodes, and then propose an algorithm for fragment-aware virtual network reconfiguration (FA-VNR). The FA-VNR algorithm selects the virtual nodes to be migrated and the target physical nodes from two aspects: resource fragment degree and embedding cost. The simulation results show that the FA-VNR algorithm can improve the performance of the online VNE algorithm, and is superior to the existing VN reconfiguration algorithm.

## Network model and problem description

### Physical network

The physical network is modeled as a weighted undirected graph *G*^*p*^ = (*N*^*p*^,*E*^*p*^), where *N*^*p*^ is the set of physical nodes, *E*^*p*^ is the set of physical links. For a given physical node *n*^*p*^ ∈ *N*^*p*^, we take *cpu*(*n*^*p*^) and *loc*(*n*^*p*^) to denote the available CPU resource and location information of *n*^*p*^. Similarly, for a given physical link *e*^*p*^ ∈ *E*^*p*^, we take *b*(*e*^*p*^) to denote the available bandwidth of *e*^*p*^.

### Virtual network

For the online VNE problem, the VNs that need to be embedded are a series of VN requests that arrive in a time series. For the *m*^*th*^ arriving VN request, we use $VNR_{m}=(G_{m}^{v},t_{a},t_{e})$ to represent, where $G_{m}^{v}$ is the virtual network topology, and *t*_*a*_ and *t*_*e*_ are the arrival time and end time of the VN request, respectively. The virtual network topology is also represented by a weighted undirected graph $G_{m}^{v}=(N_{m}^{v},E_{m}^{v})$, where $N_{m}^{v}$ is the set of virtual nodes and $E_{m}^{v}$ is the set of virtual links. For a given virtual node $n^{v}\in N_{m}^{v}$, we take *cpu*(*n*^*v*^) and *loc*(*n*^*v*^) to denote the required CPU resource and location information of *n*^*v*^. For a given virtual link $e^{v}\in E_{m}^{v}$, we take *b*(*e*^*v*^) to denote the required bandwidth of *e*^*v*^. When the VN reconfiguration starts each time, the VN requests that have been embedded and have not left are denoted by $\bar{G}=(\bar{N},\bar{E})$, where $\bar{N}$ and $\bar{E}$ respectively represent the set of embedded virtual nodes and the set of embedded virtual links.

### VN reconfiguration

**Fig 1:**
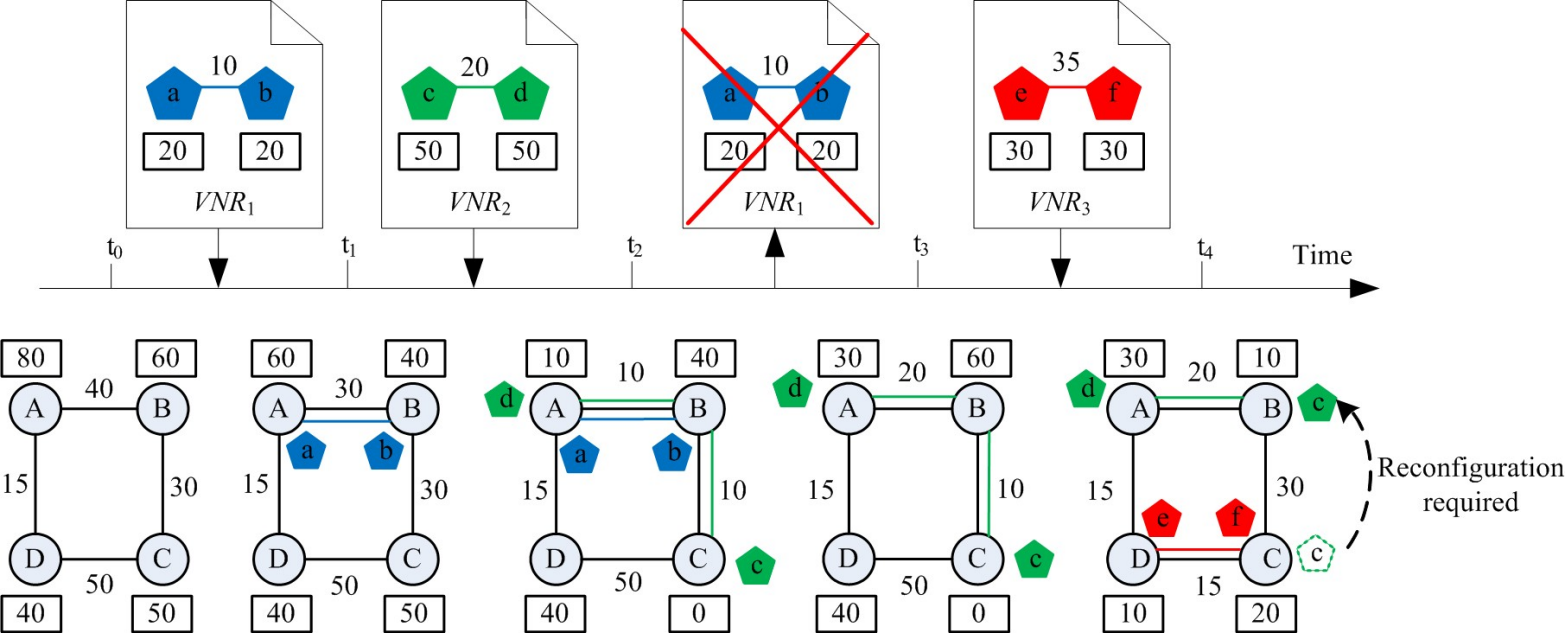
An example of VN reconfiguration.

VN reconfiguration is the remapping of the VNs that have been allocated resources on the physical network. Through proper reconfiguration, it can not only improve the ability of the physical network to accept the future VN requests, but also reduce the embedding cost of the embedded VNs. Fig 1 depicts a simple example of the VN reconfiguration. In the figure, the attribute of any node *n*, i.e. *cpu*(*n*), is depicted in the rectangle next to the node and the number next to any link *e* represents the attribute of the link, i.e. *b*(*e*). The VN reconfiguration procedure can be demonstrated in Fig 1: (I) At time t_0_, the VN request has not arrived yet, and no VN is hosted on the physical network. (II) At time t_1_, the VN request *VNR*_1_ has arrived, its node embedding scheme is {a→A,b→B}, and the link embedding scheme is {(a,b)→(A,B)}. (III) At time t_2_, the VN request *VNR*_2_ has arrived, its node embedding scheme is {d→A,c→C}, and the link embedding scheme is {(d,c)→(A,B,C)}. (IV) At time t_3_, the VN request *VNR*_1_ has left, and only the *VNR*_2_ is hosted on the physical network. (V) At time t_4_, the VN request *VNR*_3_ has arrived. At this time, if the virtual node c in *VNR*_2_ is migrated from the physical node C to the physical node B, it can not only release 20 units of bandwidth resources, but also enable *VNR*_3_ to be embedded successfully; otherwise, if no reconfiguration operation is performed, the *VNR*_3_ will not be embedded successfully due to limited bandwidth requirement. Therefore, for the online VNE algorithm, selecting an appropriate VN reconfiguration algorithm can improve its performance.

### Reconfiguration objectives

The objective of VN reconfiguration is to further improve the performance of the existing VNE algorithms. Therefore, it is similar to the objectives of the existing VNE algorithm [6,19]. In this paper, the performance of VN reconfiguration algorithm is evaluated by using the acceptance ratio and revenue to cost ratio.

The acceptance ratio is defined as the ratio of the number of successfully embedded VN requests to the total number of VN requests within a certain period of time, as shown in Eq (1).

$$\eta (Τ)=\underset{T\to \infty }{\lim}\frac{\sum_{t=0}^{T}VNR_{s}}{\sum_{t=0}^{T}VNR_{a}}$$ (1)

Where *VNR*_*s*_ represents the VN requests which have been successfully embedded during the time from 0 to *T*, *VNR*_*a*_ represents the VN requests which have arrived during the time from 0 to *T*.

The embedding revenue of $VNR_{m}=(G_{m}^{v},t_{a},t_{e})$ at time *t* is defined as Eq (2).

$$R(G_{m}^{v},t)=\sum_{n^{s}\in N_{m}^{v}}cpu(n^{s})+\sum_{e^{st}\in E_{m}^{v}}b(e^{st})$$ (2)

The embedding cost of $VNR_{m}=(G_{m}^{v},t_{a},t_{e})$ at time *t* is defined as Eq (3).

$$C(G_{m}^{v},t)=\sum_{n^{s}\in N_{m}^{v}}\sum_{n^{i}\in N^{p}}x_{i}^{s}\cdot cpu(n^{s})+\sum_{e^{st}\in E_{m}^{v}}\sum_{e^{ij}\in E^{p}}y_{ij}^{st}\cdot b(e^{st})$$ (3)

Where $x_{i}^{s}$ is a binary variable denoting the embedding relation between the virtual node *n*^*s*^ and the physical node *n*^*i*^, and $y_{ij}^{st}$ is a binary variable denoting the embedding relation between the virtual link *e*^*st*^ and the physical link *e*^*ij*^. $x_{i}^{s}$ is 1 if the virtual node *n*^*s*^ is embedded onto the physical node *n*^*i*^, and 0 otherwise. $y_{ij}^{st}$ is 1 if the virtual link *e*^*st*^ is embedded onto the physical link *e*^*ij*^, and 0 otherwise.

The revenue to cost ratio is defined as the ratio of the embedding revenue to the embedding cost in a certain period of time, as shown in Eq (4).

$$\varphi (T)=\underset{T\to \infty }{\lim}\frac{\sum_{t=0}^{T}\sum_{G_{m}^{v}\in VNR_{s}}R\left(G_{m}^{v},t\right)}{\sum_{t=0}^{T}\sum_{G_{m}^{v}\in VNR_{s}}C\left(G_{m}^{v},t\right)}$$ (4)

## Mathematical model of VN reconfiguration

### Definition of node’s fragment degree

Currently, typical VN reconfiguration algorithms [13–18], usually select virtual nodes (or virtual links) that need to be reconfigured only according to the load strength of the physical nodes (or the physical links). However, these algorithms do not consider the node resources and their adjacent link resources together, and have some sidedness. Based on the existing researches, this paper defines the physical node fragment degree to better select the virtual nodes and their adjacent virtual links that need to be reconfigured.

In VNE, performance metrics such as latency, path length, and stress level are commonly considered to improve the quality of service (QoS) of the virtual networks; while to increase the economical profit of the physical network, it is necessary to consider the metrics such as acceptance ratio and revenue to cost ratio [10,20]. In this paper, the purpose of introducing the FA-VNR algorithm into VNE process is to further improve the economic benefits of the physical network. Therefore, when defining the fragment degree of a physical node, we only consider the CPU resource consumption of the physical node and the bandwidth resources consumption of its adjacent links. Consequently, for any physical node *n*^*i*^, the definition of fragment degree is shown in Eq (5).

$$FD(n^{i})=\alpha \cdot NS(n^{i})+\beta \cdot ES(n^{i})$$ (5)

Where *NS*(*n*^*i*^) is the CPU resource load strength of *n*^*i*^, *ES*(*n*^*i*^) is the adjacent link resource load strength of *n*^*i*^, *α* is the weight coefficient of *NS*(*n*^*i*^), and *β* is the weight coefficient of *ES*(*n*^*i*^). The definitions of *NS*(*n*^*i*^) and *ES*(*n*^*i*^) are shown in Eq (6) and Eq (7), respectively.

$$NS(n^{i})=\frac{\sum_{N_{m}^{v}\in \bar{N}}\sum_{n^{s}\in N_{m}^{v}}x_{i}^{s}\cdot cpu(n^{s})}{cpu(n^{i})}$$ (6)

$$ES(n^{i})=\frac{\sum_{e^{ij}\in E(n^{i})}\sum_{E_{m}^{v}\in \bar{E}}\sum_{e^{st}\in E_{m}^{v}}y_{ij}^{st}\cdot b(e^{st})}{\sum_{e^{ij}\in E(n^{i})}b(e^{ij})}$$ (7)

In Eq (7), *E*(*n*^*i*^) represents the adjacent link set of physical node *n*^*i*^.

### ILP formulation

For the VN reconfiguration problem, on the one hand, the fragmented resources in the physical network should be reduced as much as possible to improve the acceptance ratio of VN requests. On the other hand, in order to increase the revenue to cost ratio of the physical network, the embedding cost of the VNs that have been embedded should be reduced as much as possible. Therefore, in this paper, the following multi-objective integer linear programming formulation for VN reconfiguration is constructed.

Objectives:

$$\min\;\sum_{n^{i}\in N^{p}}f(n^{i})$$ (8)

$$\max\;\sum_{G_{m}^{v}\in \bar{G}}\left[C\left(G_{m}^{v},rb\right)-C\left(G_{m}^{v},ra\right)\right]$$ (9)

Constraints:

$$\forall n^{i}\in N^{p},\sum_{N_{m}^{v}\in \bar{N}}\sum_{n^{s}\in N_{m}^{v}}x_{i}^{s}\cdot cpu(n^{s})\leq cpu(n^{i})$$ (10)

$$\forall N_{m}^{v}\in \bar{N},\forall n^{s}\in N_{m}^{v},\forall n^{i}\in N^{p},x_{i}^{s}\cdot dis(loc(n^{s}),loc(n^{i}))\leq D(n^{s})$$ (11)

$$\forall N_{m}^{v}\in \bar{N},\forall n^{i}\in N^{p},\sum_{n^{s}\in N_{m}^{v}}x_{i}^{s}\leq 1$$ (12)

$$\forall N_{m}^{v}\in \bar{N},\forall n^{s}\in N_{m}^{v},\sum_{n^{i}\in N^{p}}x_{i}^{s}=1$$ (13)

$$\forall e^{ij}\in E^{p},\sum_{E_{m}^{v}\in \bar{E}}\sum_{e^{st}\in E_{m}^{v}}y_{ij}^{st}\cdot b(e^{st})\leq b(e^{ij})$$ (14)

$$\forall E_{m}^{v}\in \bar{E},\forall e^{st}\in E_{m}^{v},\forall n^{i}\in N^{p},\sum_{n^{j}\in N^{p}}y_{ij}^{st}-\sum_{n^{j}\in N^{p}}y_{ji}^{st}=x_{i}^{s}-x_{i}^{t}$$ (15)

$$\forall N_{m}^{v}\in \bar{N},\forall n^{s}\in N_{m}^{v},\forall n^{i}\in N^{p},x_{i}^{s}\in \{0,1\}$$ (16)

$$\forall E_{m}^{v}\in \bar{E},\forall e^{st}\in E_{m}^{v},\forall e^{ij}\in E^{p},y_{ij}^{st}\in \{0,1\}$$ (17)

In Eq (8), when the fragment degree of the physical node *n*^*i*^ is greater than the threshold value *FD*_*th*_, the value of *f*(*n*^*i*^) is 1; otherwise it is 0. In Eq (9), $C\left(G_{m}^{v},rb\right)$ represents the embedding cost of the virtual network $G_{m}^{v}$ before reconfiguration, and $C\left(G_{m}^{v},ra\right)$ represents the embedding cost of the virtual network $G_{m}^{v}$ after reconfiguration. The Eq (10) indicates that the available CPU resources of the physical node should not be less than the CPU resource requirements of the virtual nodes it hosts. The Eq (11) indicates that the Euclidean distance between the physical node and its hosted virtual node should meet the requirements of the virtual node for the distance. The Eq (12) indicates that any physical node can only host at most one virtual node in the same VN request. The Eq (13) indicates that any virtual node can only be embedded onto one physical node. The Eq (14) indicates that the available bandwidth of any physical link should not be less than the bandwidth requirement of the virtual links it hosts. The Eq (15) indicates that any virtual link should be embedded onto a valid physical path. The Eqs (16) and (17) represent the range of values for variables $x_{i}^{s}$ and $y_{ij}^{st}$.

## FA-VNR algorithm design

Due to the fact that multi-objective integer linear programming is NP-hard, obtaining the optimal solution of VN reconfiguration problem is NP-hard. When the problem size is small, tools such as GLPK and CPLEX [21,22] can be used for exact solution. When the problem size is large, it is difficult to find the optimal solution within a limited time. To solve this problem, a heuristic algorithm named FA-VNR is designed in this section. It answers the following key questions: (1) When does it need to perform VN reconfiguration? (2) Which virtual nodes and their adjacent links need to be migrated? (3) Where should the virtual nodes and their adjacent links be migrated? (4) How to carry out the migration? The main steps of the FA-VNR algorithm are as follows:

**Step 1:** Determine whether the VN reconfiguration algorithm need to be started. For VN reconfiguration algorithms, the choice of trigger condition is very important. When the VN reconfiguration algorithm triggers too frequently, it will take a lot of reconfiguration time. On the other hand, when the VN reconfiguration algorithm triggers too rarely, the fragment resources and bottleneck resources in the physical network cannot be well reduced. In this paper, a simple and practical periodic VN reconfiguration mechanism is adopted, that is, a VN reconfiguration is performed at a certain interval of time *T*_*r*_.

**Step 2:** Construct the set of physical nodes *S*^*p*^ that need to be reconfigured. For online VNE, the remaining CPU resources and link resources in physical network are dynamically changing over time. When the remaining resources of the physical network are less, the fragment degrees of all nodes are relatively large; when there are many remaining resources in the physical network, the fragment degrees of all nodes are relatively small. Therefore, this paper determines the set of physical nodes that need to be reconfigured by selecting a dynamic node fragment threshold. The node fragment threshold is defined as shown in Eq (18).

$$FD_{th}=(1-\rho )\cdot \frac{\sum_{n^{i}\in N^{p}}FD(n^{i})}{\left|N^{p}\right|}+\rho \cdot \underset{n^{i}\in N^{p}}{\max}FD(n^{i})$$ (18)

Where *ρ*(0≤*ρ*≤1) is a proportional coefficient that is used to adjust the ratio of the mean value of the node fragment degree and the maximum value of the node fragment degree to the node fragment threshold. In this paper, *ρ* = 0.1 is set in the simulation process.

Select the physical nodes with fragment degrees larger than the threshold value *FD*_*th*_ and store them in the physical node set *S*^*p*^, in which the physical nodes need to be reconfigured.

**Step 3:** Construct the set of virtual nodes *S*^*v*^ that need to be migrated. When migrating a virtual node, not only the virtual node but also its adjacent virtual links must be migrated. Therefore, when selecting a virtual node that needs to be embedded, it is necessary to consider minimizing the fragment degree of the physical node that host the virtual node on the one hand, and on the other hand, it is necessary to consider minimizing the embedding cost of the virtual node and its adjacent virtual links. For this purpose, for virtual node *n*^*v*^, we define the cost to revenue ratio of the virtual node and its adjacent virtual links as shown in Eq (19).

$$CR(n^{v})=\frac{\sum_{e^{v}\in E(n^{v})}hops(e^{v})\cdot b(e^{v})}{\sum_{e^{v}\in E(n^{v})}b(e^{v})}$$ (19)

Where *E*(*n*^*v*^) represents the set of adjacent virtual links of the virtual node *n*^*v*^, and *hops*(*e*^*v*^) represents the hops of the physical path which host the virtual link *e*^*v*^.

The process of selecting virtual nodes hosted on each physical node in *S*^*p*^ is divided into the following two steps: the first step is to sort the virtual nodes by the *CR*(*n*^*v*^) values in descending order; the second step is to construct the set of virtual nodes to be migrated with the principle of migrating as few virtual nodes as possible. Based on above, the pseudo code of the algorithm for constructing the set of virtual nodes to be migrated is given by **Algorithm 1**.

**Algorithm 1** The algorithm for constructing the set of virtual nodes to be migrated

**Input:** Physical node set *S*^*p*^

**Output:** Virtual node set *S*^*v*^

1. **for** *k* = 1:|*S*^*p*^| **do**

2.         Calculate *CR*(*n*^*v*^) values of the virtual nodes that are hosted by the *k*^*th*^ physical node $S_{k}^{p}$ in *S*^*p*^

3.         Sort the virtual nodes that are hosted by $S_{k}^{p}$ in descending order of *CR*(*n*^*v*^), and record the result into *VNL*

4.         **for** *i* = 1:|*VNL*| **do**

5.             Calculate $FD(S_{k}^{p})$ of $S_{k}^{p}$ after migrating the first *i* virtual nodes in the *VNL*

6.             **if** $FD(S_{k}^{p})\leq FD_{th}$ **then**

7.                 Save the first *i* virtual nodes in *VNL* into *S*^*v*^

8.                 **break**

9.             **end if**

10.         **end for**

11. **end for**

12. **return** *S*^*v*^

**Step 4:** Sort the virtual nodes in *S*^*v*^. Considering that migrating a virtual node with a large *CR*(*n*^*v*^) value is preferred, more physical network resources can be released, which is conducive to the migration of the subsequent virtual nodes. Therefore, in this step, the virtual nodes in *S*^*v*^ are arranged in descending order of *CR*(*n*^*v*^) values and the virtual nodes with high *CR*(*n*^*v*^) values are migrated preferentially.

**Step 5:** Construct the candidate target physical node set *T*^*p*^. For each virtual node in *S*^*v*^, the candidate target physical nodes are selected according to the following principles: (1) the target physical node cannot be a physical node that needs to be reconfigured; (2) any two virtual nodes in the same VN request cannot be embedded onto a same physical node; (3) the available CPU resource of the target physical node should be larger than the CPU resource requirement of the virtual node to be migrated; (4) the target physical node needs to satisfy the position constraint.

**Step 6:** Select the virtual node migration scheme. After the virtual node is migrated to the target physical node, the shortest path algorithm is used to re-embed the adjacent virtual links of the virtual node. When the virtual node $S_{k}^{v}$ in the virtual network $G_{m}^{v}$ is migrated from the physical node *n*^*i*^ to the physical node *n*^*j*^, the amount of reduction in fragment degree and the amount of reduction in resource consumption of the physical network are shown in Eq (20) and Eq (21), respectively.

$$\delta_{FD}=\left[FD(n^{j})+FD(n^{i})\right]-\left[\bar{FD(n^{j})}+\bar{FD(n^{i})}\right]$$ (20)

$$\delta_{C}=C(G_{m}^{v},rb)-C(G_{m}^{v},ra)$$ (21)

In Eq (20), *FD*(*n*^*j*^) represents the fragment degree of *n*^*j*^ before the migration of the virtual node; *FD*(*n*^*i*^) represents the fragment degree of *n*^*i*^ before the migration of the virtual node; $\bar{FD(n^{j})}$ represents the fragment degree of *n*^*j*^ after the migration of the virtual node; $\bar{FD(n^{i})}$ represents the fragment degree of *n*^*i*^ after the migration of the virtual node.

When choosing the virtual node migration scheme, the fragment degree of the physical network should be reduced first and the resource cost of the physical network should be reduced as much as possible. Therefore, the following three principles are defined to determine the migration scheme of virtual node.

- The reduction of the fragment degree is greater than 0, that is, *δ*_*FD*_>0;
- Minimize the consumption of physical network resources, that is, *δ*_*C*_≥0;
- The *δ*_*FD*_ and *δ*_*C*_ of all the migration schemes that satisfy the above conditions (I) and (II) are normalized, then the normalized *δ*_*FD*_ and *δ*_*C*_ are added to obtain *δ*, and the migration scheme with the largest *δ* value is selected as the final migration scheme.

Based on above, the pseudo code of the algorithm for selecting the virtual node migration scheme is given by **Algorithm 2**.

**Algorithm 2** The algorithm for selecting the virtual node migration scheme

**Input:** Virtual node $S_{k}^{v}$, the candidate physical node set *T*^*p*^

**Output:** The migration scheme of $S_{k}^{v}$

1.     **for** *i* = 1:|*T*^*p*^| **do**

2.         Migrate $S_{k}^{v}$ to the *i*^*th*^ physical node $T_{i}^{p}$ in *T*^*p*^

3.         **if** *δ*_*FD*_>0 && *δ*_*C*_≥0 **do**

4.             Save $T_{i}^{p}$ into the available migration scheme set *Can*

5.         **end if**

6.     **end for**

7.     **if** *Can*⊂∅ **then**

8.         **return** Virtual node $S_{k}^{v}$ migration failed

9.     **else**

10.         **for** *j* = 1:|*Can*| **do**

11.                 Normalize the *δ*_*FD*_ and *δ*_*C*_ of the *j*^*th*^ physical node in *Can*, and add the two to get *δ*

12.         **end for**

13.         Select the physical node with the largest *δ* value in *Can* as the migration scheme of $S_{k}^{v}$

14.     **end if**

15.     **return** The migration scheme of $S_{k}^{v}$

**Step 7:** Skip to step 5 and continue if the migration of all virtual nodes is not completed.

## Experimental environment setup and results analysis

### Experimental environment settings

In order to evaluate the performance of VNE algorithms, one has to perform appropriate simulations. The ALEVIN [23] is an excellent simulation tool for evaluating VNE algorithms. Unfortunately, it lacks the ability to evaluate online VNE approaches with reconfiguration [23]. Based on the simulation framework of ALEVIN, we developed a simulator using MATLAB to evaluate the performance of our online FA-VNR algorithms.

Similar to [6], we randomly generate the topologies of physical network and virtual networks by using GT-ITM tool [24,25]. We assume that the VN requests arrival in a Poisson process. The lifetime of each VN request follows an exponential distribution. The detailed parameters of the physical network and virtual networks used in our simulation are shown in Table 1.

**Table 1:** Simulation assumptions and parameters.

Parameters	Physical network	Virtual networks
Number of nodes	100	[2, 10]
Node CPU resource	[50, 100]	[0, *M*_*cpu*_]
Node position	Distributed in the scope *L*×*L* = 100×100	Distributed in the scope *L*×*L* = 100×100
Number of Links	500	Each pair of nodes is interconnected with a probability of 0.5
Link bandwidth resource	[50, 100]	[0, *M*_*bw*_]
VN request arrival rate	-	*Pois*{0.05}
VN request life time	-	*Expo*{1000}

In Table 1, [*x*_min_,*x*_max_] denotes a uniform distribution between *x*_min_ and *x*_max_. *Pois*{*p*} and *Expo*{*e*} stand for the Poisson and exponential distributions with mean *p* and *e*, respectively.

In our simulation, we set the position constraint to *D*(*n*^*s*^) = 40, the VN reconfiguration period to 500 time units, and test the performance of the FA-VNR algorithm from both the acceptance ratio and the revenue to cost ratio. The RTA-MAX algorithm proposed in [6] is used as the basic algorithm, and the FA-VNR algorithm proposed in this paper and the TA-VNR algorithm proposed in [18] are used to reconfigure the embedded VNs periodically. The detail of the compared algorithms is list in Table 2.

**Table 2:** Algorithm comparison.

Notation	Description
RTA-MAX	It means that only the RTA-MAX algorithm is used in the VNE process and no reconfiguration algorithm is used.
RTA-MAX+FA-VNR	It means that the FA-VNR algorithm is periodically used in the VNE process.
RTA-MAX+TA-VNR	It means that the TA-VNR algorithm, which is the topology-awareness VN reconfiguration algorithm proposed in [18], is periodically used in the VNE process.

### Results analysis

#### Performance comparison

**Fig 2:**
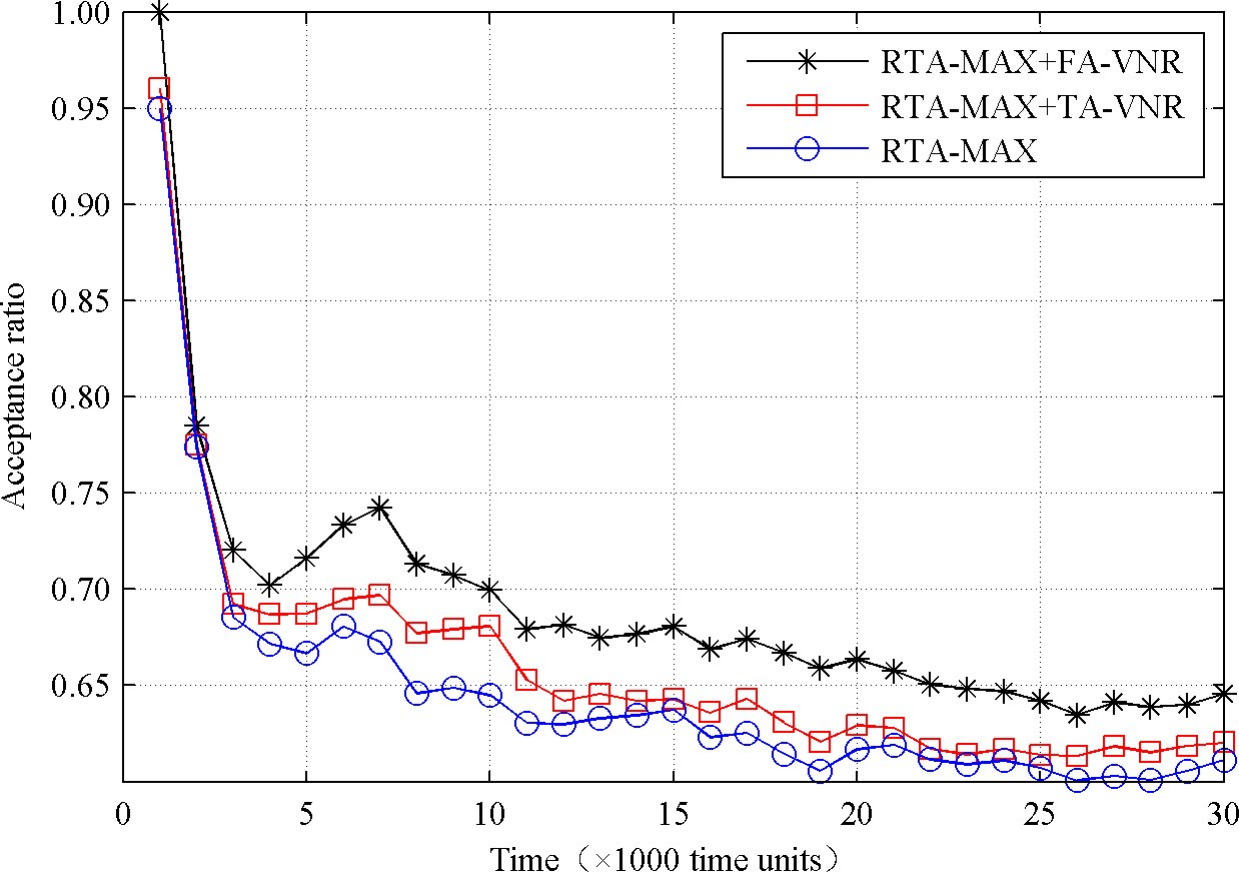
Acceptance ratio comparison.

**Fig 3:**
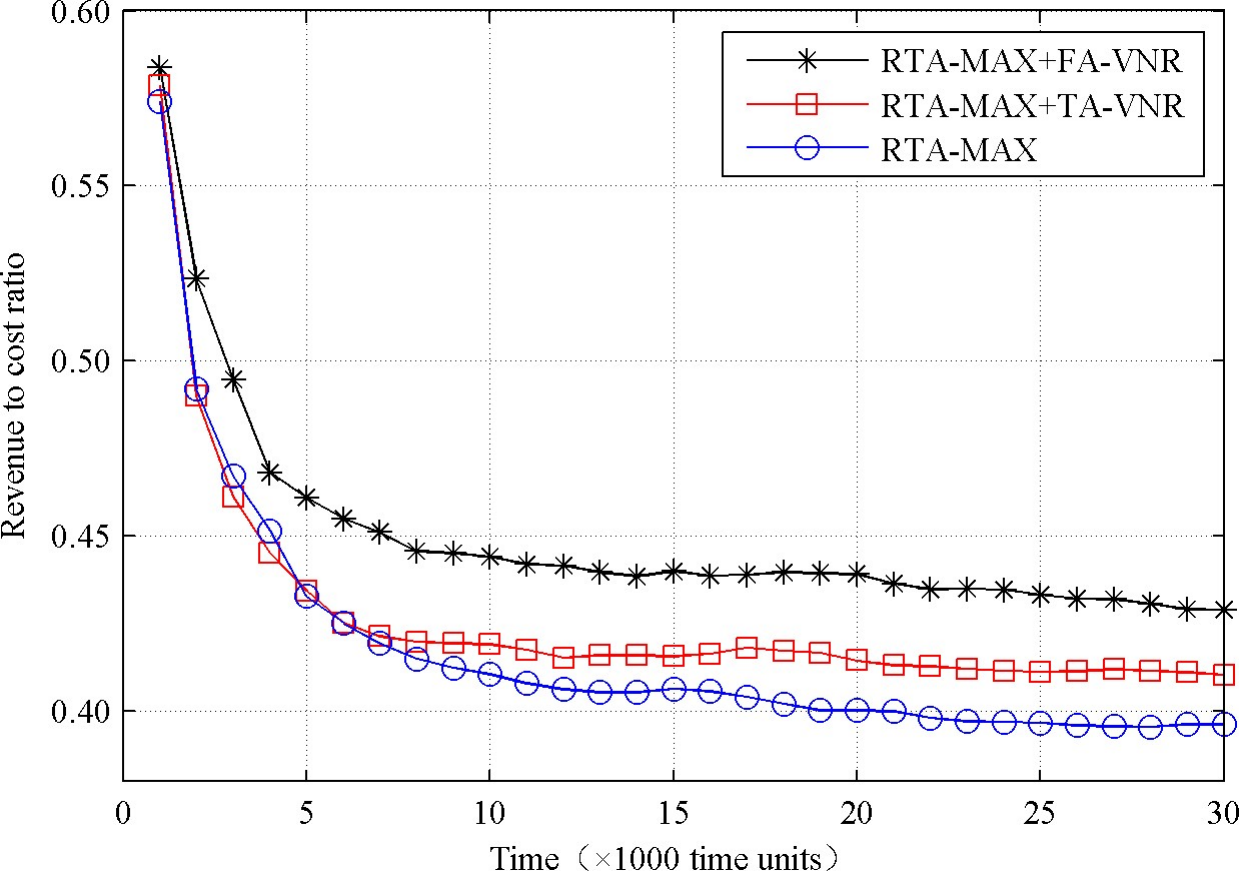
Revenue to cost ratio comparison.

To compare the performance of the three algorithms, we set the values of parameters *M*_*cpu*_ and *M*_*bw*_ to 50, and give the comparison results in Figs 2 and 3.

From Fig 2, we can see that the proposed reconfiguration algorithm improves the acceptance ratio and is superior to the current VN reconfiguration algorithm. When the simulation time is 30000 time units, the acceptance ratios of RTA-MAX+FA-VNR, RTA-MAX+TA-VNR and RTA-MAX algorithms are 64.56%, 62.03%, and 61.14%, respectively. The former increased by 4.08% and 5.59% respectively compared with the latter two. The relationship between the acceptance ratio and time is mainly related to the arrival number and departure number of the VN requests. At the initial stage, the number of arrived VN requests is less and the available resources of the physical network are more abundant, so the acceptance ratio is higher. As the simulation time continues, the number of arrived VN requests gradually increases, and the available resources of the physical network gradually decrease, so the acceptance ratio rapidly declines. When the simulation time is to a certain extent, the arrival rate and the departure rate of the VN requests tend to be stable, and the available resources of the physical network tend to be stable, so the acceptance ratio tends to be stable. The reasons why the RTA-MAX+FA-VNR algorithm has the highest acceptance ratio are as follows. First, the FA-VNR algorithm dynamically adjusts the fragment degrees of the physical nodes through the migration mechanism to improve the load balancing of the physical network. Secondly, the FA-VNR algorithm reduces the embedding cost of the embedded VNs through reconfiguration and releases some physical link resources.

As can be seen from Fig 3, the reconfiguration algorithms optimize the revenue to cost ratio of the physical network, and the effect of the proposed algorithm is more obvious. When the simulation time is 30000 time units, the revenue to cost ratios of RTA-MAX+FA-VNR, RTA-MAX+TA-VNR and RTA-MAX algorithms are 42.88%, 41.02%, and 39.60%, respectively. The former increased by 4.53% and 8.28% respectively compared with the latter two. Compared with other algorithms, the RTA-MAX+FA-VNR algorithm not only improves the average embedding revenue of the physical network, but also reduces the average embedding cost of the physical network, so it has the highest revenue to cost ratio. In the initial stage of simulation, the available resources of the physical network are abundant, resulting in better embedding schemes for the VN requests, so the revenue to cost ratios of all the three algorithms are higher. After a period of simulation, the arrival rate and the departure rate of the VN requests tend to be stable, and the available resources of the physical network also tend to be stable, resulting in the stability of the revenue to cost ratios of all the three algorithms.

#### Evaluation with different resource requirement of VN requests

**Fig 4:**
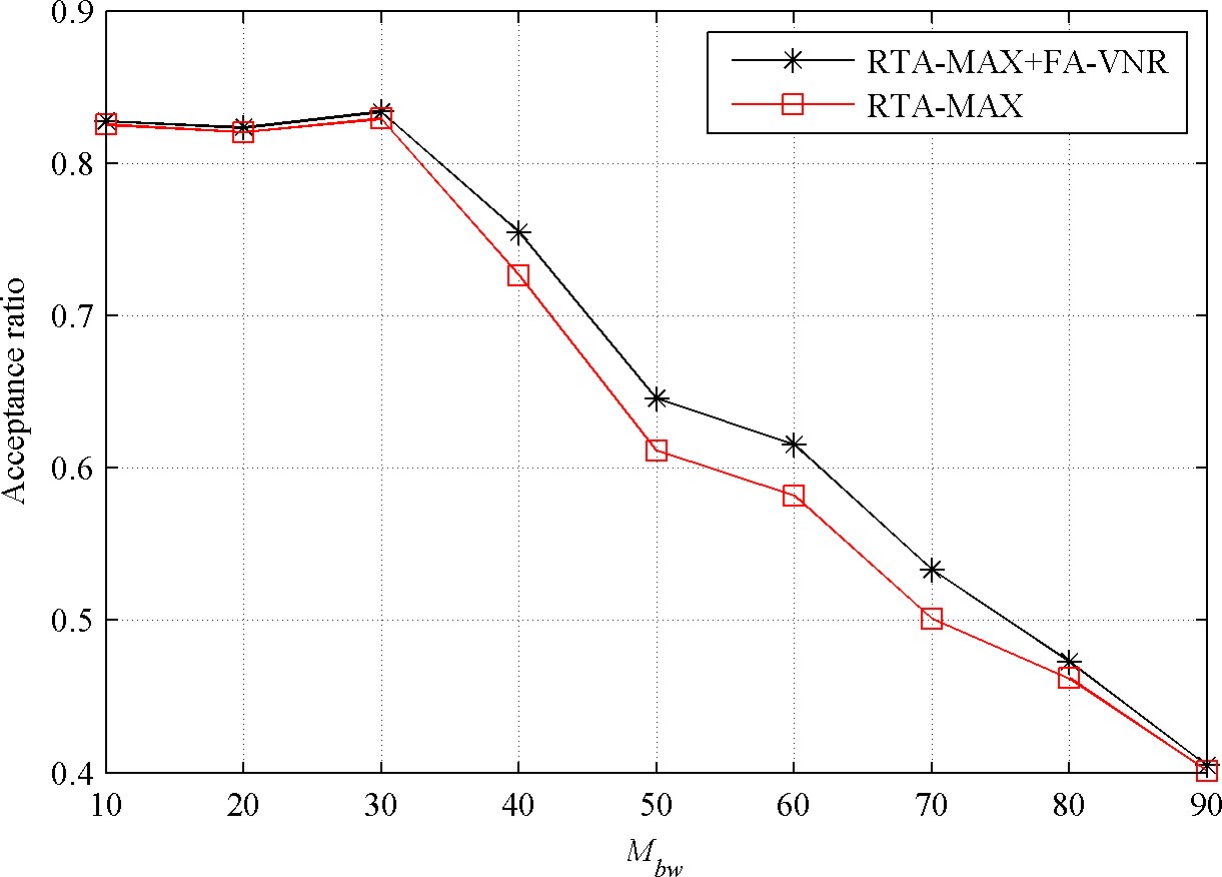
Acceptance ratio with increasing *M*_*bw*_.

**Fig 5:**
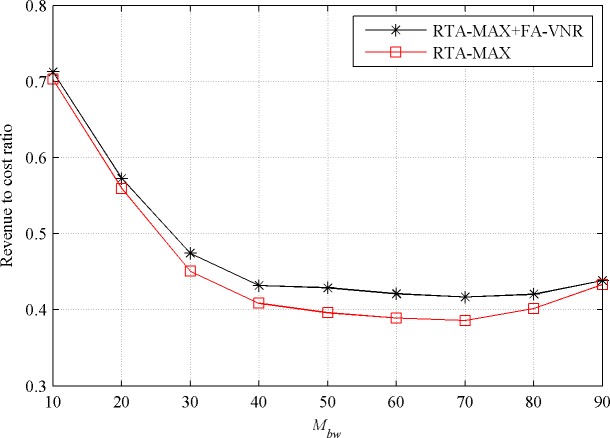
Revenue to cost ratio with increasing *M*_*bw*_.

In order to further verify the performance of the proposed reconfiguration algorithm, we investigate the influence of the bandwidth resource requirement and CPU resource requirement of the VN requests on the performance of it. When the value of *M*_*bw*_ increases from 10 to 90 while keeping the value of *M*_*cpu*_ at 50, the effects of the FA-VNR algorithm on the performance of the RTA-MAX algorithm in the stable state are shown in Figs 4 and 5.

From Fig 4, we can see that the proposed reconfiguration algorithm FA-VNR improves the acceptance ratio of the basic algorithm RTA-MAX. When parameter *M*_*bw*_ is less than 30, the optimization effect of the FA-VNR algorithm is not obvious. This is because when the maximum bandwidth requirement of the VN requests is small, the embedding failure of the VN requests is mainly caused by the failure of node embedding. When *M*_*bw*_ is greater than 40 and less than 70, the FA-VNR algorithm has a significant improvement in the acceptance ratio of the RTA-MAX algorithm, and the acceptance ratio of RTA-MAX+FA-VNR (0.6372 on average) is about 5.29% higher than that of RTA-MAX (0.6052 on average). When the parameter *M*_*bw*_ is greater than 80, the maximum bandwidth requirement of the virtual links is close to the maximum available bandwidth resource of the physical links. By reconfiguration, the physical link load cannot be changed well, so the FA-VNR algorithm has only a slight improvement in the acceptance ratio of the RTA-MAX algorithm.

From Fig 5, we can see that the proposed reconfiguration algorithm FA-VNR improves the revenue to cost ratio of the basic algorithm RTA-MAX. With the increase of *M*_*bw*_, the improvement effect of the FA-VNR algorithm on the revenue to cost ratio of the RTA-MAX algorithm increases first and then decreases. When *M*_*bw*_ is greater than 40 and less than 70, the FA-VNR algorithm has a significant improvement in the revenue to cost ratio of the RTA-MAX algorithm, and the revenue to cost ratio of RTA-MAX+FA-VNR (0.4246 on average) is about 7.52% higher than that of RTA-MAX (0.3949 on average).

**Fig 6:**
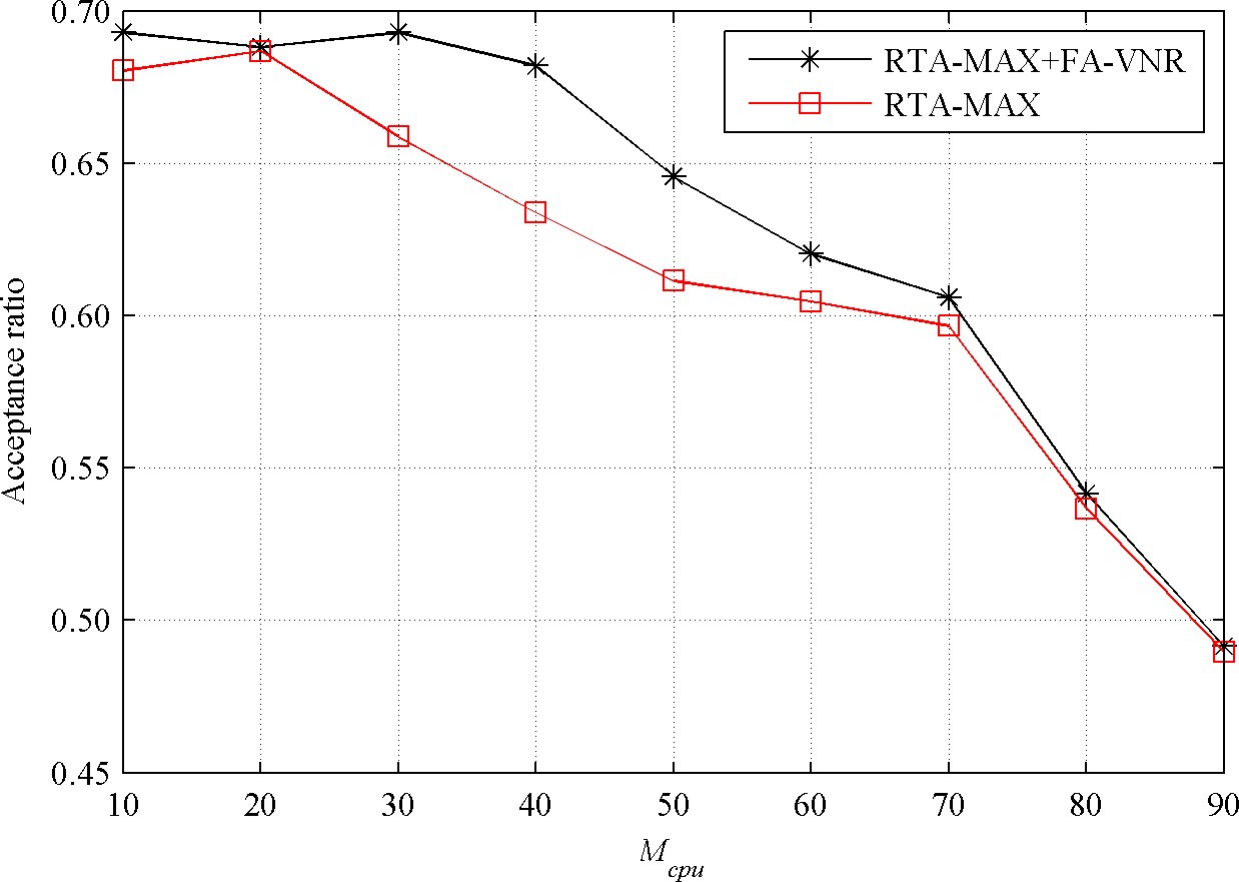
Acceptance ratio with increasing *M*_*cpu*_.

**Fig 7:**
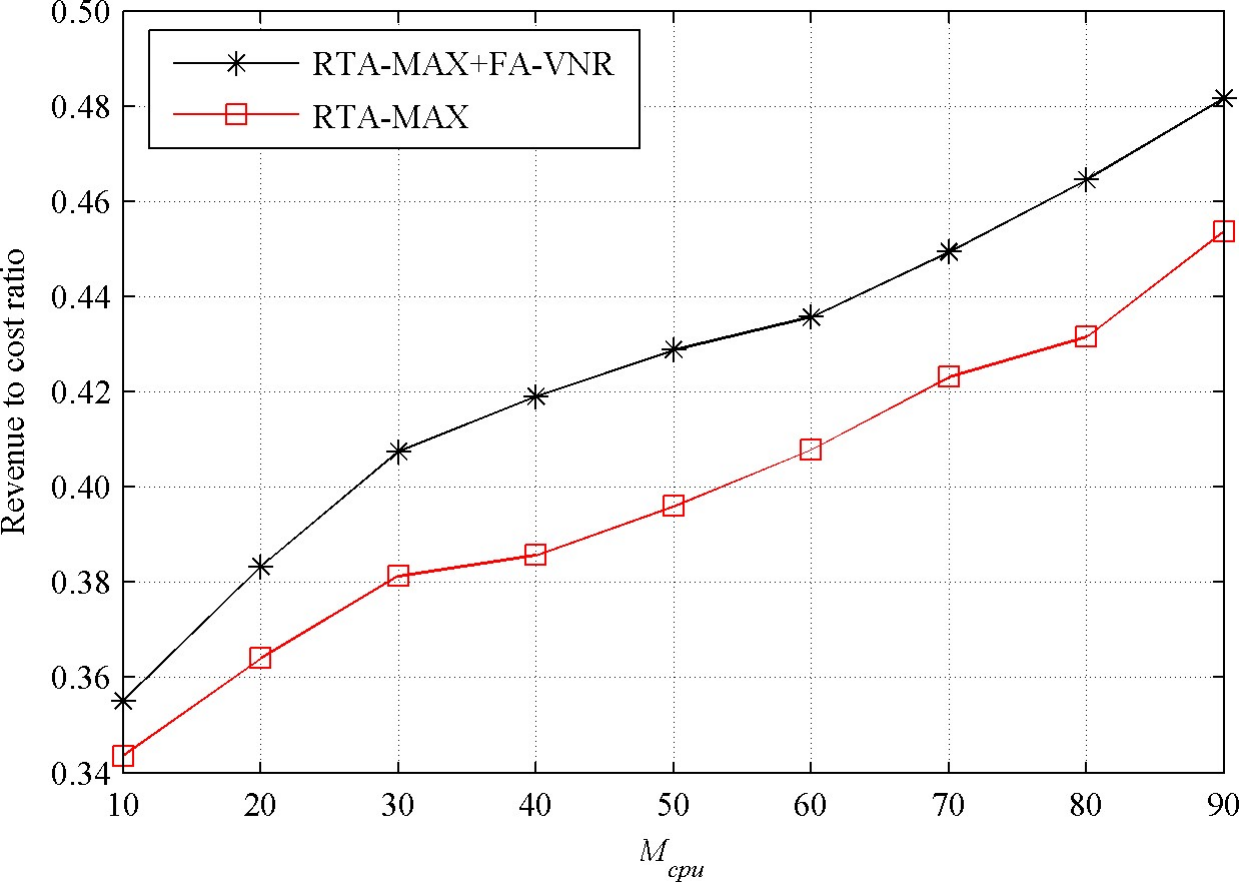
Revenue to cost ratio with increasing *M*_*cpu*_.

When the value of *M*_*cpu*_ increases from 10 to 90 while keeping the value of *M*_*bw*_ at 50, the acceptance ratios and revenue to cost ratios of the RTA-MAX+FA-VNR and RTA-MAX algorithms in the stable state are shown in Figs 6 and 7, respectively.

From Fig 6, we can see that when the value of *M*_*cpu*_ is smaller (less than 20) or larger (greater than 60), the improvement effect of the FA-VNR algorithm on the acceptance ratio of the RTA-MAX algorithm is not obvious. When the *M*_*cpu*_ value is more than 30 and less than 50, the improvement effect is more obvious, and the acceptance ratio of RTA-MAX+FA-VNR (0.6735 on average) is about 6.12% higher than that of RTA-MAX (0.6346 on average).

From Fig 7, we can see that under all simulation conditions, the FA-VNR algorithm can significantly improve the revenue to cost ratio of the RTA-MAX algorithm, and the revenue to cost ratio of RTA-MAX+FA-VNR (0.4250 on average) is about 6.64% higher than that of RTA-MAX (0.3985 on average).

## Conclusion

In this paper, in order to improve the performance of the online VNE algorithm, a method to measure the fragment degree of the physical node is defined first, then an integer linear programming model for VN reconfiguration is constructed and a heuristic VN reconfiguration embedding algorithm FA-VNR is proposed. By periodically executing the FA-VNR algorithm, the fragment degrees of the physical nodes can be reduced and the embedding cost of the embedded VNs can be reduced, thus the acceptance ratio and the revenue to cost ratio of the basic algorithm can be improved. Through extensive simulation experiments, it is shown that when the maximum resources requirement of the VN requests are too large or too small, the FA-VNR algorithm has no obvious effect on the improvement of the basic algorithm RTA-MAX; when the maximum resources requirement of the VN requests are moderate, the FA-VNR algorithm can obviously improve the performance of the basic algorithm RTA-MAX. However, the latency of the physical and virtual networks has not been considered in this paper. To extend our work, we intend to introduce metrics such as latency, path length, and stress level into VN reconfiguration to provide QoS guaranteed virtual networks.

## Supporting information

S1 Fig Physical network topology. This figure depicts the topology of the physical network in the simulations.
(TIF)

S1 File Initial data. This document contains the initial data in the simulations.
(ZIP)
