# Supplementary figures and images for: An algorithm for fragment-aware virtual network reconfiguration
Source: PLoS One. 2018 Nov 21;13(11):e0207705. doi: 10.1371/journal.pone.0207705 (PMC6248979; doi:10.1371/journal.pone.0207705)

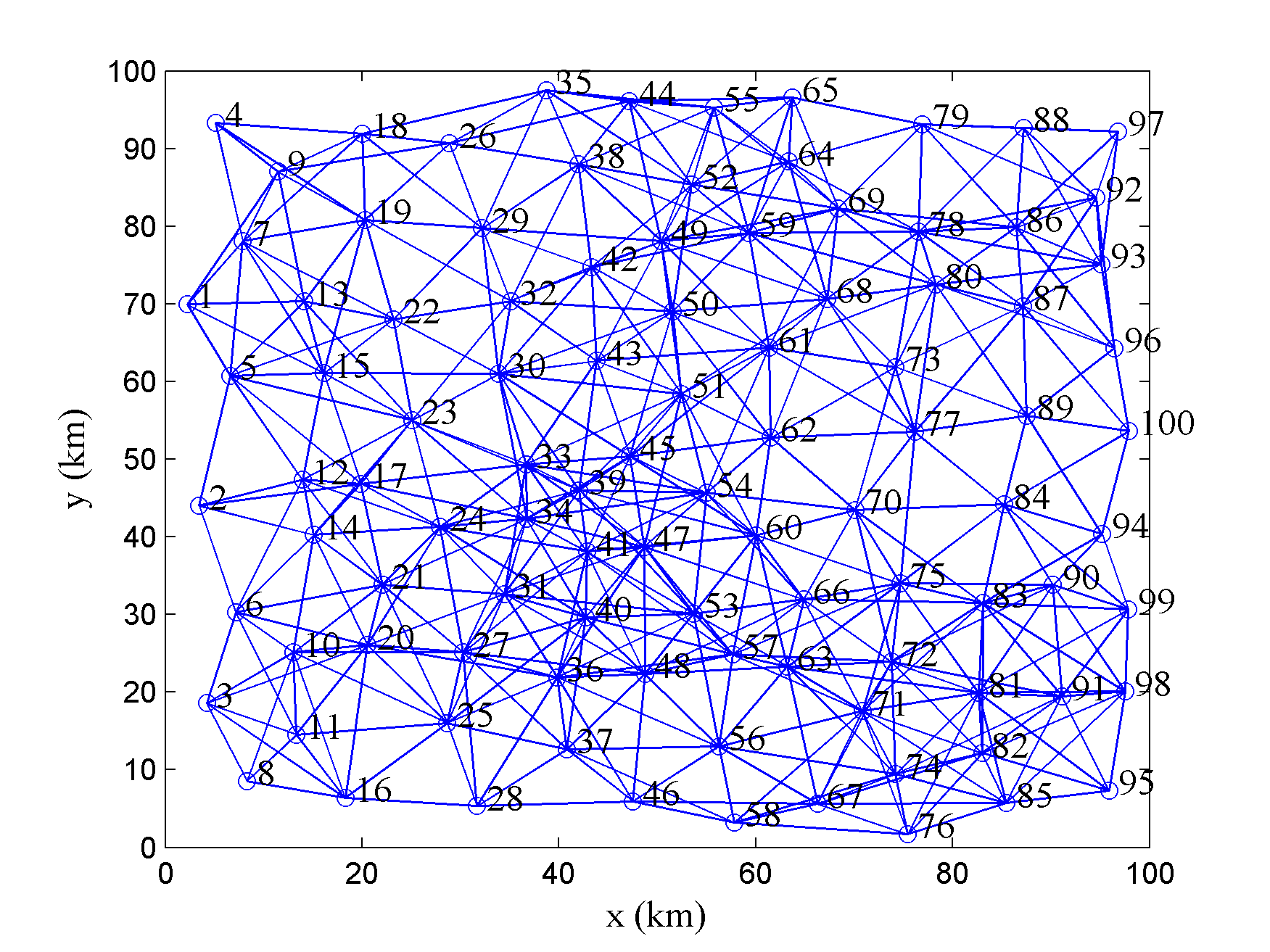

Supplement: S1 Fig — This figure depicts the topology of the physical network in the simulations. (TIF) [file pone.0207705.s001.tif]
